# To what extent do chickens suffer when gassed with CO_2_?

**DOI:** 10.3389/fvets.2025.1719226

**Published:** 2026-01-12

**Authors:** Jenny L. Mace, Andrew Knight

**Affiliations:** 1 Centre for Ethics, Philosophy and Public Affairs, University of St Andrews, St Andrews, United Kingdom; 2 Mace Animal Welfare, Dunfermline, United Kingdom; 3 School of Veterinary Medicine, College of Environmental and Life Sciences, Murdoch University, Murdoch, WA, Australia; 4 School of Environment and Science, Griffith University, Nathan, QLD, Australia; 5 Faculty of Health and Wellbeing, University of Winchester, Sparkford Road, Winchester, United Kingdom

**Keywords:** chicken gas slaughter, chicken welfare, CO2 slaughter, CO2 stunning, gas slaughter

## Introduction

On 9th June 2025, in an apparent world first, undercover footage of chickens being gassed to death, in the UK in 2024, was publicly released (1). The footage depicts a CO_2_ gas pit system within which crated chickens are mechanically lowered into increasing concentrations of CO_2_ [(2), p. 82]. Specifically, the footage captured the CO_2_ stunning of “spent” laying hens (1)—laying hens of roughly 1.5 years of age who are no longer sufficiently productive to be of laying value to the egg industry. According to Carbstrong (1), this includes hens from farms certified with the UK higher welfare label of RSPCA Assured. *The Independent* covered this news story and featured commentary from an RSPCA Assured spokesperson who had seen a longer nine-minute clip (3). According to *The Independent*, the RSPCA spokesperson dismissed *any* welfare concerns arising from the footage, stating: “the birds in the footage were already unconscious and were not in pain”. The following short article seeks to highlight the flaws within this blanket statement, and to suggest what the RSPCA could instead be advocating, as a leading UK animal welfare organisation and a provider of higher welfare labelling (4).

## The onset of loss of consciousness in chickens during CO_2_ gassing

CO_2_ gas stunning/slaughter has become the main method of stunning/slaughtering chickens in the UK (5). This followed a transition away from inverting and shackling chickens by their feet to proceed towards a water bath electrical stunning system. Whilst a move away from inverting/shackling the chickens is welcome, it is far from clear if distress caused by CO_2_ is actually significantly less than the distress from handling, inversion, and shackling.

High concentration CO_2_ is aversive to chickens. Their tracheal chemoreceptors facilitate perception of this as an irritant when above certain concentrations [(6), p. 2]. Further aversion can result from a sense of air hunger or breathlessness, hypoxia (low blood oxygen), hypercapnia (high blood CO_2_), subsequent acidosis of blood and bodily tissues, and nervous system dysfunction [(7), p. 30–31; (6)]. Chickens can demonstrate an awareness of CO_2_ at concentrations even lower than 5% [(8), p. 452; (9), p. 181]. When they cannot escape from the gas, distress behaviours commence from around 6% and can continue up to 30% concentrations (assuming incremental increases) or to the point of unconsciousness—whichever comes first [(9), p. 182; (10)]. Distress behaviours can include gasping, headshaking, high-pitched/shrieking vocalisations, jumping, and manic/strenuous escape attempts [(11), p. 59, 72; (9), p. 182]. Loss of posture then follows whereby heads are down/limp and chickens can no longer stand [(6), p. 7]. Loss of posture is often used as a proxy for loss of consciousness [(11), p. 27]. Any of the aforementioned behaviours evident *post* loss of posture are not typically deemed welfare concerns as the chickens are normally considered unconscious.

For chicken stunning/slaughter, the UK legal requirement is for biphasic exposure to CO_2_, with a maximum concentration of 40% CO_2_ in the first phase, and a higher CO_2_ concentration in the second phase. However, there is not a requirement for incremental increases to the CO_2_ concentration (e.g., see [(12), Part 5, Point 30; (13), Annex I, Ch. 1, Table 3]). Additionally, it should be noted that, as of February 2026, the government's official Guidance document (14) lists high concentration CO_2_ for standard poultry slaughter (except for geese and ducks) as legal, while legislation (13) forbids it apart from for emergency poultry slaughter. This inconsistency must be urgently rectified as many stakeholders will rely on the Guidance documents to explain legislation. In contrast, the RSPCA Assured label requires *both* a biphasic approach *and* incremental increases to the CO_2_ concentration. This means there must be a first stunning phase whereby the CO_2_ concentration increases gradually. Additionally, in the first phase, the CO_2_ concentration must never exceed an average maximum of 30% [(2), p. 82]. The first phase brings about loss of consciousness in chickens; the second phase ensures their death in concentrations of CO_2_ over 40%. Whilst concentrations of CO_2_ 40% and over are considered particularly aversive to chickens, the downside of this more incremental approach is the longer time period required for chickens to become unconscious. Evidence suggests an average of 59.2 seconds until loss of consciousness transpires in chickens placed directly into near 40% CO_2_ concentrations [(6), p. 9], whilst an average of four minutes may be required in the biphasic, incremental approach adopted by the RSPCA [(9), p. 183]. It remains unclear whether shorter bouts of more intense pain/distress are worse or better for welfare than longer less intense bouts of pain/distress (15).

## Why the RSPCA's blanket statement was inaccurate

According to *The Independent*, the RSPCA spokesperson stated that the chickens appearing in the footage were unconscious and so not experiencing any pain (3). However, as outlined, CO_2_ stunning is not instantaneous. This is dissimilar to the stunning processes the public is somewhat used to for other farmed animal species such as cattle and sheep. Stunning of cattle and sheep typically involves the application of a captive bolt pistol to the brain. This is instantaneous if the equipment is correctly maintained and operated (16).

However, in the video footage in question, the process of losing consciousness has only *begun*, at best, for many of the chickens in the footage, as most are only *in the midst* of losing posture. It is most helpful to distinguish this stage by naming it as *losing balance* or *control* as Gerritzen et al. also do [(9), p. 182]. Thus, awareness and the ability to feel pain/distress will remain *during* this process. This includes awareness of and sensitisation to the aforementioned effects of CO_2_ inhalation. Moreover, debate and doubt remain over whether the culmination of this process—the full loss of posture—is even a reliable indicator of loss of consciousness (e.g., [(8), p. 454; (11), p. 27]). If being lenient to the RSPCA spokesperson, their statement may apply to *some* of the chickens who have *fully* lost their posture and are no longer flailing around. Crucially however, this is not true for the majority of chickens depicted in the footage. The RSPCA spokesperson's statement seems unaligned with the RSPCA's own science department's advice [(10), p. 16] and appears to ignore the experience of distress more broadly, with a narrow focus on pain.

There is evidence of endogenous opioid release in times of stress in mammals and avians. However, as described by Ferdousi and Finn (17) and Baker et al. (18), this does *not* necessarily result in animals (including humans) feeling *no* pain or distress. Furthermore, it is stressor-specific, constituting a physiological coping mechanism in *some* cases of stress. The authors note that (1) stress can also elicit the opposite physiological response, thereby *increasing* sensitivity to pain, and (2) there are multiple other sources of distress besides pain. This indicates the importance of observing a holistic set of species-specific indicators of pain and distress (19). The harmful and non-instantaneous effects of CO_2_ stunning are also likewise known for a number of other species, including rats (20) and pigs (21). Indeed, there is mounting pressure to ban the gassing of pigs with CO_2_ (22).

## What the RSPCA could be advocating

The RSPCA Assured label is intended to guarantee a higher level of welfare than the legal baseline or mainstream forms of farming and slaughter (23). However, as (1) CO_2_ stunning/slaughter is now the predominant method in the UK (5), and (2) there is no certainty regarding shorter bouts of more intense pain/distress being worse than longer bouts of more moderate pain/distress, it is reasonable to ask: what superior welfare for chickens at slaughter is the RSPCA Assured label guaranteeing today? The RSPCA could require a meaningful and reliable higher welfare alternative for chickens slaughtered under its label. There *are* higher welfare alternatives available that are feasible with use of existing infrastructure. Namely, replacing CO_2_ with inert gases could secure higher welfare [(6), p. 2, (18)]. This is because inert gases are believed to be non-aversive to chickens [(11), p. 28]. Specifically, the inert gas argon is recommended as it, like CO_2_, is heavier than air. This means it could easily be kept contained within the gas pits using current infrastructure. However, if alternatives to CO_2_ are being considered for new operations without infrastructure in place, then nitrogen, as an alternative inert gas, may be a preferred option as it is slightly cheaper than argon. The RSPCA's own science department encourages use of inert gases [(10), p. 16], so it seems incongruent that the RSPCA Assured label does not require this.

## Conclusions

In summary, if loss of posture is accepted as an indicator of unconsciousness, the chickens in the footage in question are either *beginning* to lose or *in the process* of losing consciousness. This is because the loss of posture is not yet complete for most chickens in the footage. Hence, it cannot be asserted they have *already* lost consciousness and no longer feel pain, as an RSPCA spokesperson has claimed. The RSPCA could assist in securing the replacement of CO_2_ stunning/slaughter with argon or nitrogen stunning/slaughter to live up to their higher welfare labelling claims.
