# Correction: To what extent do chickens suffer when gassed with CO_2_?

**DOI:** 10.3389/fvets.2026.1800875

**Published:** 2026-02-24

**Authors:** Jenny L. Mace, Andrew Knight

**Affiliations:** 1 Centre for Ethics, Philosophy and Public Affairs, University of St Andrews, St Andrews, United Kingdom; 2 Mace Animal Welfare, Dunfermline, United Kingdom; 3 School of Veterinary Medicine, College of Environmental and Life Sciences, Murdoch University, Murdoch, WA, Australia; 4 School of Environment and Science, Griffith University, Nathan, QLD, Australia; 5 Faculty of Health and Wellbeing, University of Winchester, Sparkford Road, Winchester, United Kingdom

**Keywords:** chicken gas slaughter, chicken welfare, CO2 slaughter, CO2 stunning, gas slaughter

There was an error in Section “The onset of loss of consciousness in chickens during CO_2_ gassing”, third paragraph. The paragraph stated:

“For chicken stunning/slaughter, the UK legal requirement is for exposure to a minimum concentration of 40% CO_2_. A biphasic approach to CO_2_ gassing refers to a first phase comprising a lower, and a second phase comprising a higher concentration of CO_2_. However, there is neither a requirement for a biphasic approach nor for incremental increases to the CO_2_ concentration (e.g., see [(12), Part 5, Point 30; (13), Annex I, Ch. 1, Table 3; (14)]).”

Its corrected version appears below:

“For chicken stunning/slaughter, the UK legal requirement is for biphasic exposure to CO_2_, with a maximum concentration of 40% CO_2_ in the first phase, and a higher CO_2_ concentration in the second phase. However, there is not a requirement for incremental increases to the CO_2_ concentration (e.g., see [(12), Part 5, Point 30; (13), Annex I, Ch. 1, Table 3]). Additionally, it should be noted that, as of February 2026, the government's official Guidance document (14) lists high concentration CO_2_ for standard poultry slaughter (except for geese and ducks) as legal, while legislation (13) forbids it apart from for emergency poultry slaughter. This inconsistency must be urgently rectified as many stakeholders will rely on the Guidance documents to explain legislation”.

The original article has been updated.

